# Distinct macrophage uptake of engineered and biological particles driven by host age and sex

**DOI:** 10.1080/14686996.2025.2610875

**Published:** 2026-01-12

**Authors:** Riki Toita, Yuki Shimizu, Jeong-Hun Kang

**Affiliations:** a Molecular Biosystems Research Institute, National Institute of Advanced Industrial Science and Technology (AIST), Ikeda, Osaka, Japan; b Molecular Biosystems Research Institute, National Institute of Advanced Industrial Science and Technology (AIST), Tsukuba, Ibaraki, Japan; c National Cerebral and Cardiovascular Center Research Institute, Suita, Osaka, Japan

**Keywords:** Nanomedicines, aging, biological sex, macrophages, uptake, RNA-seq

## Abstract

Understanding how nano- and microparticles interact with biological systems is essential for advancing the biomedical applications of engineered materials. These interactions are governed not only by the physicochemical properties of the particles – such as size, shape, and surface chemistry – but also by host-specific physiological factors. However, how intrinsic host factors, particularly age and biological sex, affect interactions between immune cells and particles remains poorly understood. In this study, we systematically investigated how these intrinsic host variables influence the cellular uptake of polymeric particles by primary macrophages. Using a library of particles with controlled sizes (25, 250, and 3000 nm) and surface chemistries (unmodified, amine-, and carboxyl-functionalized), as well as with biological particles (bacteria and yeast), we compared uptake efficiencies in macrophages derived from male and female mice of various ages. We observed significant age- and sex-dependent differences in particle internalization. Transcriptomic profiling revealed differentially expressed genes related to receptor-mediated endocytosis and actin cytoskeleton remodeling, suggesting that molecular mechanisms underly these variations. Additionally, protein coronas were formed by incubating polymeric particles with autologous serum, revealing sex-dependent differences in corona composition and resulting macrophage recognition. Our findings highlight the critical interplay between engineered-material properties and host biological variability. Accordingly, this work provides key insights for the rational design of nanomaterials tailored to perform consistently across heterogeneous biological populations, thereby advancing the development of personalized nanomedicine and immunomodulatory materials.

## IMPACT STATEMENT

This study reveals how age and sex influence the macrophage uptake of particles, emphasizing the importance of host-dependent variability in designing consistent, personalized nanomedicines and advancing applications of immunomodulatory materials.

## GRAPHICAL ABSTRACT

**Figure:**
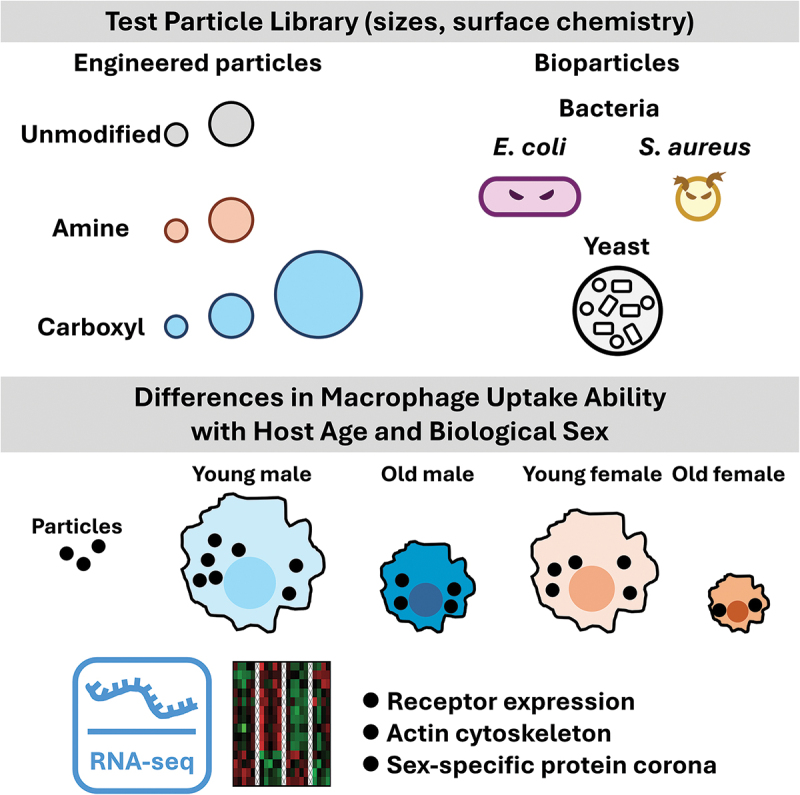

## 1. Introduction

Surface-engineered nano- and microparticles have emerged as versatile platforms for drug delivery, immune modulation, and diagnostic applications [1]. Upon systemic administration, these particles encounter a complex biological environment where their interactions with immune cells are determined not only by physicochemical parameters such as size, surface charge, shape, and composition, but also by intrinsic host-specific factors [2]. As the field moves toward personalized and population-inclusive nanomedicine, it is becoming more apparent that physiological variables such as age and biological sex can influence the biointerface [3,4]. However, despite this growing awareness, such host-related biological diversity remains underestimated in most experimental designs for evaluating particle behavior.

Among immune cell populations, macrophages are central to the recognition and clearance of foreign materials, including engineered particles and pathogens [5,6]. They also serve critical roles in innate immune defense, antigen presentation, and maintenance of tissue homeostasis [6]. Their functional capacity, however, is not static. Aging is associated with a chronic, low-grade inflammatory state, which can alter macrophage phenotype, cytokine secretion, and phagocytic behavior [7–10]. Similarly, sex differences in immune function, driven by hormonal, epigenetic, and chromosomal factors, can contribute to differential disease susceptibility and therapeutic outcomes [3,11,12]. These host-dependent immune variations likely impact how macrophages recognize and internalize foreign particles. However, few studies have examined how particle uptake is shaped by the combined effects of host age, host sex, and particle design.

An additional layer of complexity arises from the formation of a protein corona, which is a dynamic layer of adsorbed serum proteins that forms on the material surface upon contact with biological fluids [2]. The corona reshapes the biological identity of particles, thereby influencing cellular recognition and uptake. Given that serum protein composition is known to vary with age and sex [13–16], corona formation may serve as a key mediator through which host physiology alters particle – cell interactions. Nevertheless, few studies have investigated how autologous protein coronas, derived from physiologically distinct hosts, influence macrophage responses.

In this study, to address these gaps, we systematically evaluated how host age and sex influence particle uptake behavior in primary macrophages. Bone marrow-derived macrophages (BMMs) were isolated from male and female mice at three life stages, young (3‒4 months), middle-aged (12‒15 months), and old (22‒26 months). They were exposed to a panel of seven polymeric particles differing in size (25, 250, and 3000 nm) and surface chemistries (plain, amino, and carboxyl groups). To assess whether observed differences were specific to engineered materials, the BMMs were also challenged with three bioparticle types: inactivated Gram-negative bacteria, inactivated Gram-positive bacteria, and yeast cell walls (hereafter collectively referred to as bioparticles). Furthermore, to examine the role of host serum composition, autologous protein coronas were formed on polymeric nanoparticles using serum collected from the same animal donors. This aspect enabled us to evaluate whether host-specific corona signatures contribute to differences in macrophage uptake. Our results reveal that host age and sex notably influence particle phagocytosis, with outcomes shaped by both particle properties and biological background.

## 2. Materials and methods

### 2.1. Particles

A series of fluorophore-modified polystyrene spherical particles (micromer-redF; micromod Partikeltechnologie GmbH, Rostock, Germany) of different sizes (25, 250, and 3000 nm) and surface chemistries (plain, amino groups, and carboxyl groups) were used in the uptake studies. Fluorescein-labeled dead gram-negative (*Escherichia coli; E. coli*) and gram-positive bacteria (*Staphylococcus aureus; S. aureus*) and zymosan A (derived from yeast *Saccharomyces cerevisiae*) (from Thermo Fisher Scientific, Waltham, MA, U.S.A.) were used as bioparticles. Zymosan, which is a β-1,3-glucan polysaccharide, has long been used as a model particle for phagocytosis. The sizes of the particles used were confirmed using dynamic light scattering (DLS) with a Zetasizer NS (Malvern Instruments, Malvern, UK) equipped with a 633 nm laser.

### 2.2. Cellular experiments

Cell studies using mouse primary macrophages were approved by the National Institute of Advanced Industrial Science and Technology (Admission numbers: A2024-, A2025-404) and performed in accordance with the Guidelines for Animal Experiments established by the Ministry of Health, Labour and Welfare of Japan. Young (3‒4 months), middle-aged (12‒15 months), and elderly (22‒26 months) C57BL/6 mice of both sexes purchased from Japan SLC (Shizuoka, Japan) were euthanized to isolate BMMs [17]. Their ages were equivalent to 10‒12, 41‒51, and 76‒90 years-old in humans, respectively [18]. Under isoflurane anesthesia, blood was collected from the inferior vena cava of mice, and serum was prepared (used in Subsection 2.2.3). Bone marrow cells were then flushed from the femurs and cultured in RPMI-1640 medium (RPMI; Fujifilm Wako Pure Chemical) supplemented with 10% fetal bovine serum (FBS; Thermo Fisher Scientific), 1% penicillin/streptomycin (P/S; Fujifilm Wako Pure Chemical), and 40 ng/mL recombinant mouse macrophage colony stimulating factor (M-CSF; BioLegend, San Diego, CA, U.S.A.). On day 3, 5 mL of fresh media was added to the culture dishes. On day 5, the adherent cells were used as BMMs in subsequent experiments. The purity of BMMs was typically greater than 95%, as confirmed by CD11b immunostaining (Figure S1). Importantly, our previous report demonstrated that BMMs prepared using this method remain in an unpolarized, naïve state and exhibit only background levels of cytokines associated with either M1 or M2 activation. This indicates that there is no baseline bias toward M1 or M2 polarization [19]. RAW 264.7 mouse macrophages were cultured in Dulbecco’s modified Eagle’s medium (DMEM; Fujifilm Wako Pure Chemical) supplemented with 10% FBS and 1% P/S.

#### 2.2.1. Uptake study

BMMs were seeded in 24-well culture plates at an initial density of 100,000 cells/well and cultured overnight in RPMI containing 10% FBS and 40 ng/mL M-CSF. Then, 1 mL dispersions of fluorophore-labeled polymeric particles or bioparticles were added to the cultured cells at a final concentration of 50 μg/mL. At the specified time, the cells were washed three times with PBS and dissolved in lysis buffer M (Fujifilm Wako Pure Chemical). Fluorescence intensity (Ex/Em = 485/530 nm [bioparticles] and 550/590 mm [polymeric particles]) was measured using a Synergy HT plate reader (BIO-TEK Instruments Inc., Winooski, VT, U.S.A.), and then particle concentration in the lysate was calculated using a standard curve (Figures S2 and S3). The protein concentration in the lysate was determined using Protein Assay Kits (Bio-Rad Laboratories, Hercules, CA, U.S.A.). The amount of particles taken up was normalized by the cellular protein concentration in the lysate. To block the macrophage receptor with collagenous structure (MARCO), cells were treated with 1 μg/mL rat anti-mouse MARCO antibody (clone ED31; GeneTex, Irvine, CA, U.S.A.) during particle exposure, and particle uptake was subsequently assessed as described above.

#### 2.2.2. RNA-seq analysis

BMMs were seeded in six-well culture plates at an initial density of 100,000 cells/well and cultured overnight in RPMI containing 10% FBS and 40 ng/mL M-CSF. Cells were lysed in 2 mL TRIzol reagent (Thermo Fisher Scientific), and RNA was extracted using a Direct-Zol RNA MicroPrep Kit (Zymo Research, Irvine, CA, U.S.A.). Libraries were prepared for NovaSeq6000 (Illumina, CA, U.S.A.) with 2 × 150 bp paired ends. Data analysis was conducted following our previous report [20] with minor modifications. Sequencing reads were trimmed with Trimmomatic-0.39 [21] to remove adapters and low-quality bases, aligned to the Mus musculus reference genome (GRCm39) using HISAT2 (v2.2.1) [22], and quantified at the gene level with featureCounts (v2.0.2) [23] using Ensembl annotations. Principal component analysis (PCA) was performed with the iDEP 2.0 pipeline [24] to assess sample variation and potential batch effects. Batch effects detected in the initial PCA were corrected using ComBat-seq [25], and PCA was repeated to confirm their mitigation. One sample appeared as a potential outlier in the post – batch-correction PCA; it was evaluated using Mahalanobis distance [26] and excluded from downstream analyses. Differentially expressed genes (DEGs) were identified using DESeq2 [27] with a fold-change cutoff of 1.5 and an adjusted *p*-value (Benjamini – Hochberg) <0.1.

#### 2.2.3. Protein corona formation and uptake experiment

Mouse serum was prepared as described in Cellular experiments. A polymeric particle solution (1 mg/mL) was mixed with serum at a volume ratio of 1:1, and the mixture was incubated at 37°C for 1 h to form a protein corona on the particle surface [13]. To avoid replacement of the protein corona by FBS, the solution was diluted 10-fold with FBS-free DMEM, and the resulting nanoparticle dispersion was used in subsequent uptake assays. RAW 264.7 macrophages were seeded at an initial density of 100,000 cells/well in 24-well culture plates and cultured overnight in DMEM containing 10% FBS. After the culture plates were washed with PBS, 1 mL of nanoparticle dispersion (final concentration of 50 μg/mL) was added to each well. After 4 h incubation, nanoparticle uptake was measured using the method described in subsection 2.2.1.

### 2.3. Statistical analysis

All data are presented as mean ± standard error of the mean (SE). Statistical analysis of differences between means was performed using a one-way (or two-way) analysis of variance (ANOVA) followed by Tukey’s multiple comparison test using GraphPad Prism software version 10.4.1. *p* < 0.05 was considered statistically significant.

## 3. Results

### 3.1. Particle characterization

**Figure 1.**
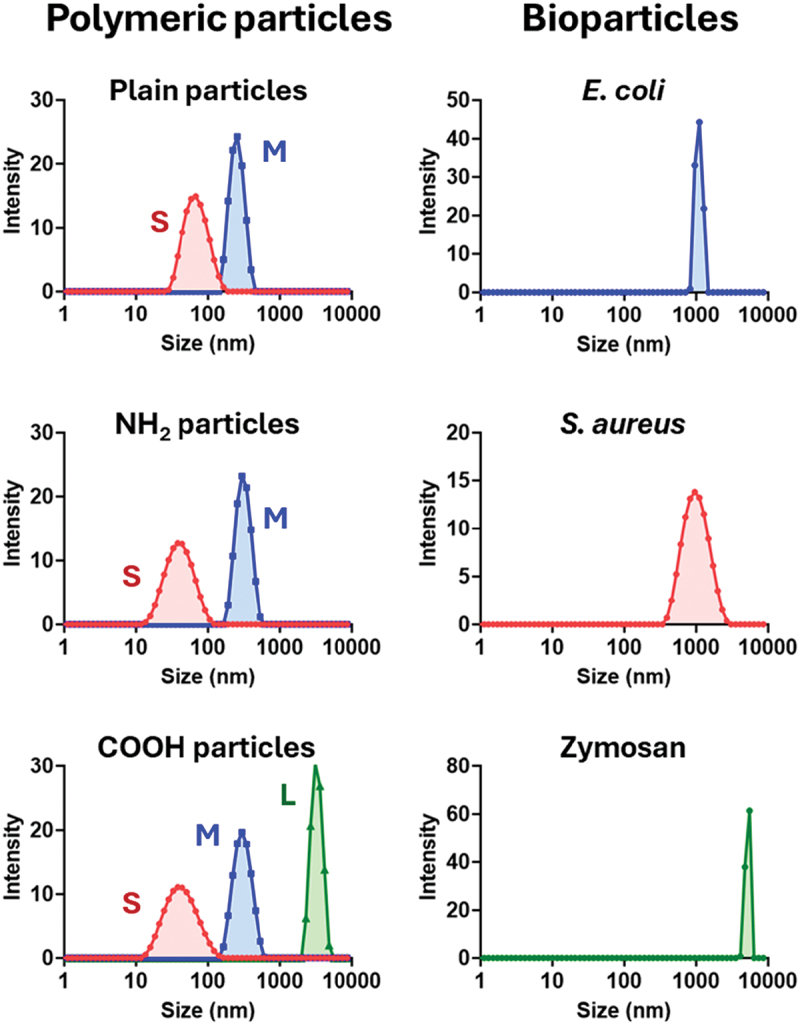
Size distributions of particles used in the present cell studies. S, small; M, medium; L, large.

Commercially available polymeric particles and bioparticles (dead bacteria [*E. coli* and *S. aureus*] and yeast cell wall [zymosan A]) were subjected to cellular uptake studies. The polymeric particles were selected based on size (tens of nm [small, S], hundreds of nm [medium, M], and thousands of nm [large, L]) and surface chemistry (plain, NH_2_, and COOH). A histogram of particle-size distribution is shown in Figure 1, and their average sizes and polydispersity indices are summarized in Table 1.

**Table 1.** Particle sizes used in uptake experiments.

Category	Name	Size (nm)^*a*^	Polydispersity^*a*^
Polymeric particles	S-plain	63 ± 0	0.12 ± 0.02
	S-NH_2_	37 ± 1	0.18 ± 0.03
	S-COOH	38 ± 0	0.19 ± 0.02
	M-plain	247 ± 3	0.03 ± 0.01
	M-NH_2_	301 ± 2	0.03 ± 0.02
	M-COOH	291 ± 3	0.08 ± 0.01
	L-COOH	3,042 ± 37	0.21 ± 0.03
Bioparticles	*E. coli*^*b*^	1,494 ± 474	0.66 ± 0.17
	*S. aureus*	847 ± 21	0.24 ± 0.02
	Zymosan A	5,392 ± 150	0.21 ± 0.01

^*a*^Data are means ± SD.

^*b*^Note that the elongated morphology of *E. coli*, unlike the near-spherical shape of the other tested particles, may have hindered cumulative DLS fitting, contributing to the larger observed variation in size and polydispersity.

### 3.2. Influence of aging and biological sex macrophage uptake capacity

#### 3.2.1. Polymeric particle uptake

**Figure 2.**
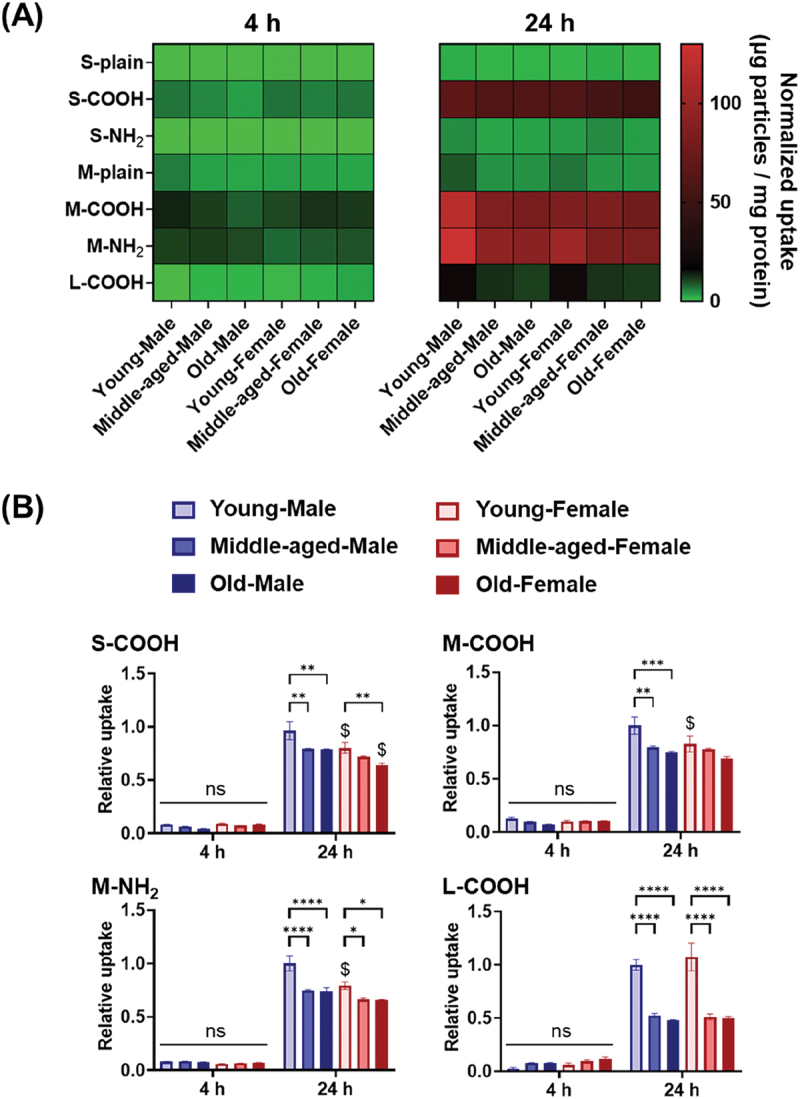
Synthetic particles uptake. (A) Heatmap of the average normalized particle uptake by bone marrow-derived macrophages (BMMs). (B) Relative particle uptake ratio, where one is the value for young male BMMs 24 h after particle addition (*n* = 3). Data are means ± SE. Statistical significance was determined by two-way ANOVA followed by Tukey’s test. *p < 0.05; **p < 0.01; ***p < 0.001; ****p < 0.0001; $*p* < 0.05 (compared with age-matched male BMM groups); ns, not significant.

Fluorescently labeled polymeric particles were exposed to BMMs, and uptake levels were measured after 4 and 24 h. After 4 h, the fluorescence intensity from particles was near the detection limit, meaning negligible uptake (Figure 2(A)). After 24 h, the BMMs were observed to have preferentially taken up S-COOH, M-COOH, M-NH_2_, and L-COOH particles, but uptake of the other particles (i.e. plain types, and S-NH_2_) showed only minor increases from the levels observed after 4 h. Thus, the presence of surface (especially negative) charges appeared to be essential for efficient uptake by BMMs, as only minor uptake was observed with neutral particles. Meanwhile, comparison of particles with similar surface chemistry (NH_2_ or COOH) also highlighted the importance of particle size, with uptake efficiency following the order : hundreds of nm > tens of nm > thousands of nm.

The magnitudes of particle uptake by BMMs were found to vary significantly with mice age and sex at 24 h, but not at 4 h (Figure 2(B)). By age, young BMMs displayed higher uptake capacity than older BMMs, regardless of sex (except for female BMMs with M-COOH particles). The trend in middle-aged BMMs was more similar to that in old BMMs than in young BMMs. The uptake of L-COOH particles decreased the most significantly with advancing age (50% compared with that of young BMMs), whereas the decrease rates for the other particles ranged 5%–30%.

In terms of sex differences, young female BMMs showed lower uptake capacities than male BMMs for S-COOH, M-COOH, and M-NH_2_ particles. No sex differences were observed in middle-aged BMMs, while in old BMMs, differential uptake was only observed for S-COOH particles, suggesting that middle-aged and old BMMs were less susceptible to sex differences than young BMMs.

#### 3.2.2. Bioparticle uptake

**Figure 3.**
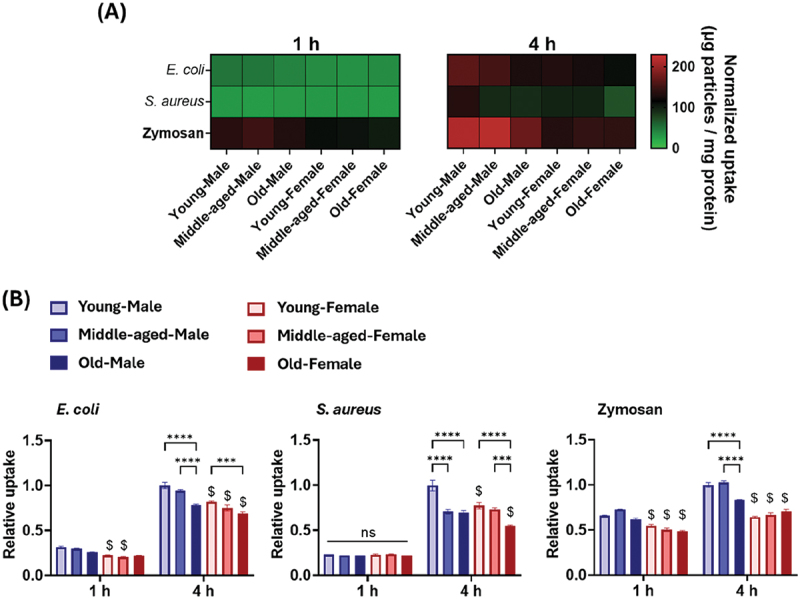
Bioparticle uptake. (A) heatmap of the average normalized particle uptake by bone marrow-derived macrophages (BMMs). (B) relative particle uptake ratio, where one is the value for uptake of young male BMMs 4 h after particle addition (*n* = 3). Data are means ± SE. Statistical significance was determined by two-way ANOVA followed by Tukey’s test. ***p < 0.001; ****p < 0.0001; $*p* < 0.05 (compared with age-matched male BMM groups); ns, not significant.

As with the polymeric particles, similar uptake experiments were conducted with bioparticles. The results showed that bioparticles tested, including *E. coli* (Gram-negative), *S. aureus* (Gram-positive) and zymosan (yeast cell walls), were taken up more rapidly and to a greater extent than the polymeric particles, allowing uptake behavior to be compared at an earlier stage (1 h; Figure 3(A) and Figure S4). The levels of bioparticle uptake increased with incubation time between 1 and 4 h. After 1 h, there was no apparent influence of age on uptake behavior in both sexes (Figure 3(B)). However, female BMMs showed less *E. coli* and zymosan uptake than male BMMs. After 4 h, the effects of age and sex became more apparent. For *E. coli* bioparticles, uptake declined with age in both sexes, and at any given age, female BMMs exhibited significantly lower uptake than males. In *S. aureus* bioparticles, the uptake ability of BMMs decreased with age in both sexes, and female BMMs exhibited lower uptake levels than their male counterparts. For zymosan, only male BMMs showed age-related reduction in uptake ability, while the lower uptake ability in females appeared to be sex-related but not age-related. The response of middle-aged BMMs fell between those of young and old BMMs and was dependent on the test substances.

#### 3.2.3. Short summary of uptake experiments

**Figure 4.**
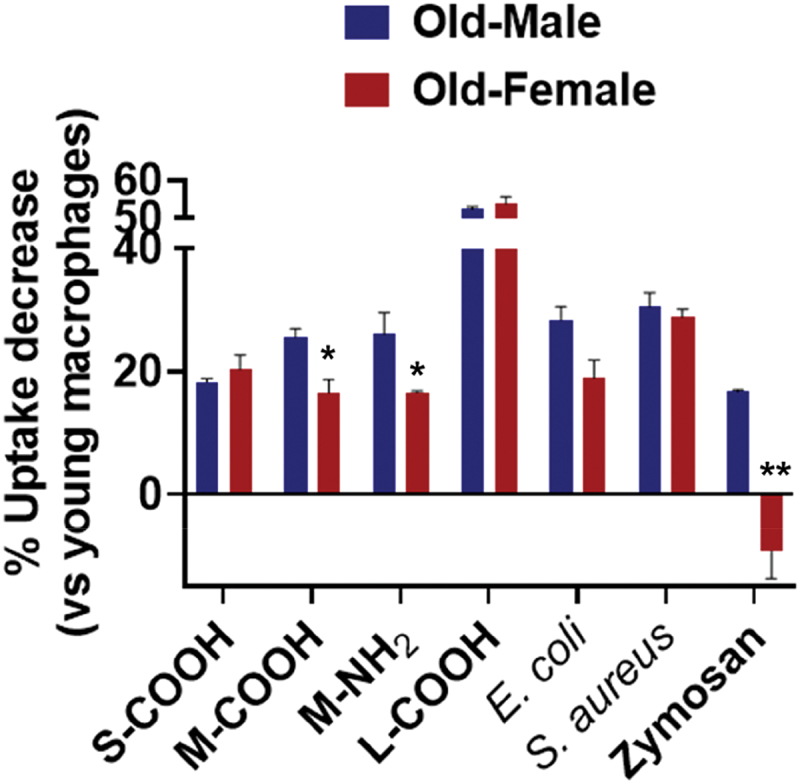
Female macrophages showed higher resistance to age-dependent decline in uptake capacity. Data are means ± SE (*n* = 3). Statistical significance was determined by paired *t*-test. *p < 0.05; **p < 0.01 (compared with male macrophages).

The results of the uptake experiments suggest that the uptake capacity of BMMs broadly declines with advanced age and/or female sex (Figures 2 and 3 and undefined). For instance, the combination of these factors causes marked differences in uptake behavior (i.e. young male BMMs vs old female BMMs). Meanwhile, age-dependent impairment in uptake of these particles (including M-COOH, M-NH_2_, and zymosan) as compared with young BMMs is smaller in the female group than in the male group (Figure 4). This suggests that female BMMs likely have a higher resistance to age-associated impairment in uptake ability.

### 3.3. Transcriptome analysis of macrophages

To better understand this differential uptake capacity, we attempted to characterize BMMs using transcriptomic analysis with RNA-seq. Because the uptake ability of middle-aged BMMs tended to be biased toward those of young or old BMMs depending on the samples tested (Figures 2 and 3), BMMs from young and old mice in both sexes were chosen for analysis. To visualize similarities in gene expression between the experimental groups, PCA was performed. Initial PCA revealed batch effects, which were corrected before further analysis (Figure S5A). A subsequent PCA indicated a potential outlier; Mahalanobis distance confirmed one old female sample as an outlier, and it was excluded from the following analysis (Figure S5B).

**Figure 5.**
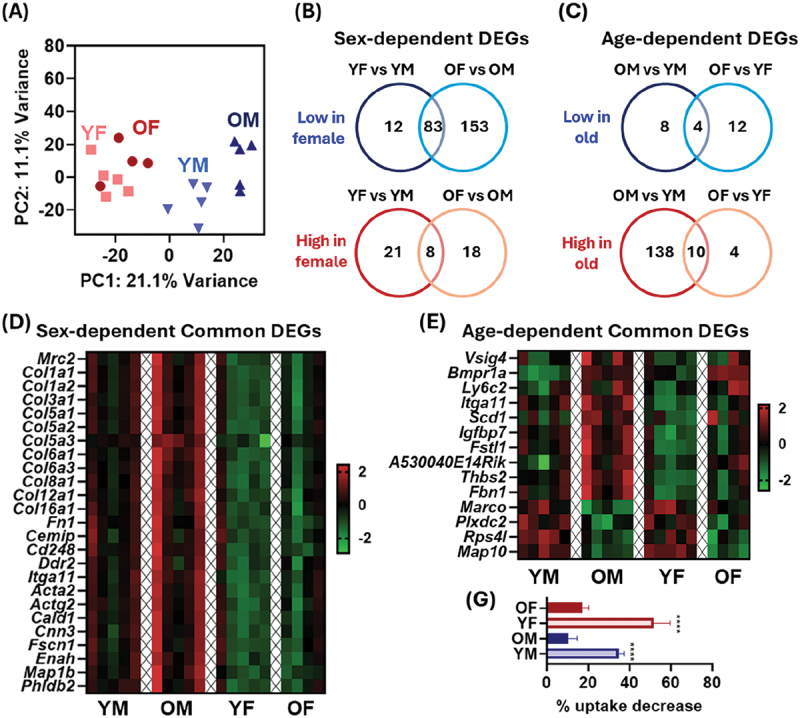
RNA-seq analysis of bone marrow-derived macrophages (BMMs). (A) Principal component analysis (*n* = 4 or 5). YM, young male BMMs; YF, young female BMMs; OM, old male BMMs; OF, old female BMMs. Venn diagram of numbers of (B) sex-dependent and (C) age-dependent differentially expressed genes (DEGs). Heatmaps of common (D) sex-dependent DEGs and (E) age-dependent DEGs. Values are *z*-score. (G) reduction in particle uptake by blocking the MARCO scavenger receptor with an anti-mouse MARCO antibody (1 μg/mL) (*n* = 5). Data are shown as the percentage reduction in uptake compared to cells without an anti-mouse MARCO antibody. Data are means ± SE. Statistical significance was determined by one-way ANOVA followed by Tukey’s test. ****p < 0.0001 (compared with sex-matched old BMM groups).

The resulting PCA suggests that gene expression patterns were clearly different between male and female groups (Figure 5(A)). More than 100 DEGs indicative of sex differences were identified between age-matched BMMs (Figure 5(B)). Among these, 91 DEGs, including 83 genes with low expression and eight genes with high expression in female BMMs compared with male BMMs, were commonly altered in both age groups. PCA also revealed distinct transcriptomes in same-sex old and young BMMs, with the male group showing greater differences than the female group (Figure 5(A)). Accordingly, more age-dependent DEGs were identified in the male group than in the female group (male: 160 DEGs, female: 30 DEGs) (Figure 5(C)). This broadly supports the difference in age-dependent impairment of uptake ability in the BMMs of old males and females (Figure 4). Fourteen genes, including four genes with reduced expression and 10 genes with increased expression in old BMMs, were identified as age-dependent DEGs shared between the male and female groups.

#### 3.3.1. Sex-dependent transcriptome and its potential influence on phagocytosis

We then focused on the common DEGs of 91 sex-dependent genes and 14 age-dependent genes. Sex-dependent DEGs potentially associated with particle uptake are presented in a heatmap (Figure 5(D)), and the complete DEGs list is shown in Table S1. During particle uptake, cells require not only receptor-mediated particle recognition but also sequential reorganization of the cytoskeleton, a process involving the actin machinery [28–32]. A transmembrane, mannose receptor family gene (*Mrc2*) was lower in female BMMs than in male BMMs. MRC1 (CD206), which belongs to the same receptor family as MRC2, promotes phagocytosis of bacteria and yeast by recognizing glycans (e.g. mannose and fucose) [33,34]. However, MRC2 has been considered to be an endocytic receptor for fragments of collagen and extracellular matrix, but not for exogenous particulate entities, including nanoparticles or microbial particles [35,36]. Accordingly, no receptor-associated DEGs were found that could explain the sex difference in particle uptake capacity. However, female BMMs showed downregulated DEGs involving extracellular matrix (ECM; collagen and fibronectin genes), ECM-binding receptors (*Cemip*, *Cd248*, *Ddr2*, *Itga11*), and reorganization of the cytoskeleton (actin reorganization and contraction [*Acta2*, *Actg2*, *Cald1*, *Cnn3*, *Fscn1*], filopodia formation [*Enah*], and actin-microtubule coordination [*Map1b*, *Phldb2*]). Among the ECM-binding receptors identified, discoidin domain receptor (DDR) and integrin α11 also function as mechanosensing receptors and affect actin-mediated cytoskeleton reorganization [37–39]. These results suggest that differences in mechanosensing and cytoskeleton organization, rather than variation in receptor expression, may account for the sex differences (as discussed later).

#### 3.3.2. Age-dependent transcriptome and its potential influence on phagocytosis

A heatmap of gene-expression levels for 14 age-dependent DEGs is displayed in Figure 5(E). MARCO belongs to a family of scavenger receptor class-A proteins essential for engulfment of a variety of synthetic particles (e.g. polymers, silicas, carbon nanotubes) and non-opsonized bacteria [40–43]. Consistent with the uptake results (Figure 4), expression of *Marco* genes decreased dramatically with age. The age-dependent DEGs also included genes associated with ECM and mechanosensing receptors (*Itga11*; higher in old BMMs) and actin-microtubule coordination (*Map10*; lower in old BMMs). Other identified DEGs are linked to, for example, inflammation regulation (*Vsig4*, *Fstl1*) and mononuclear cell markers (*Ly6c2*), and they are less relevant to the uptake process.

To further validate the functional contribution of MARCO identified by RNA-seq, an additional uptake experiment was performed in the presence of a MARCO-blocking antibody (Figure 5(G) and Figure S6). In BMMs from young male and female mice, MARCO blocking led to 35% and 52% reduction in L-COOH particle uptake, respectively. However, the inhibitory effect was substantially smaller in BMMs from old mice (~10% in old males and ~17% in old females). These results indicate that MARCO contributes to particle uptake and partially explains the age-dependent decline.

### 3.4. Protein corona modulates polymeric particle uptake

**Figure 6.**
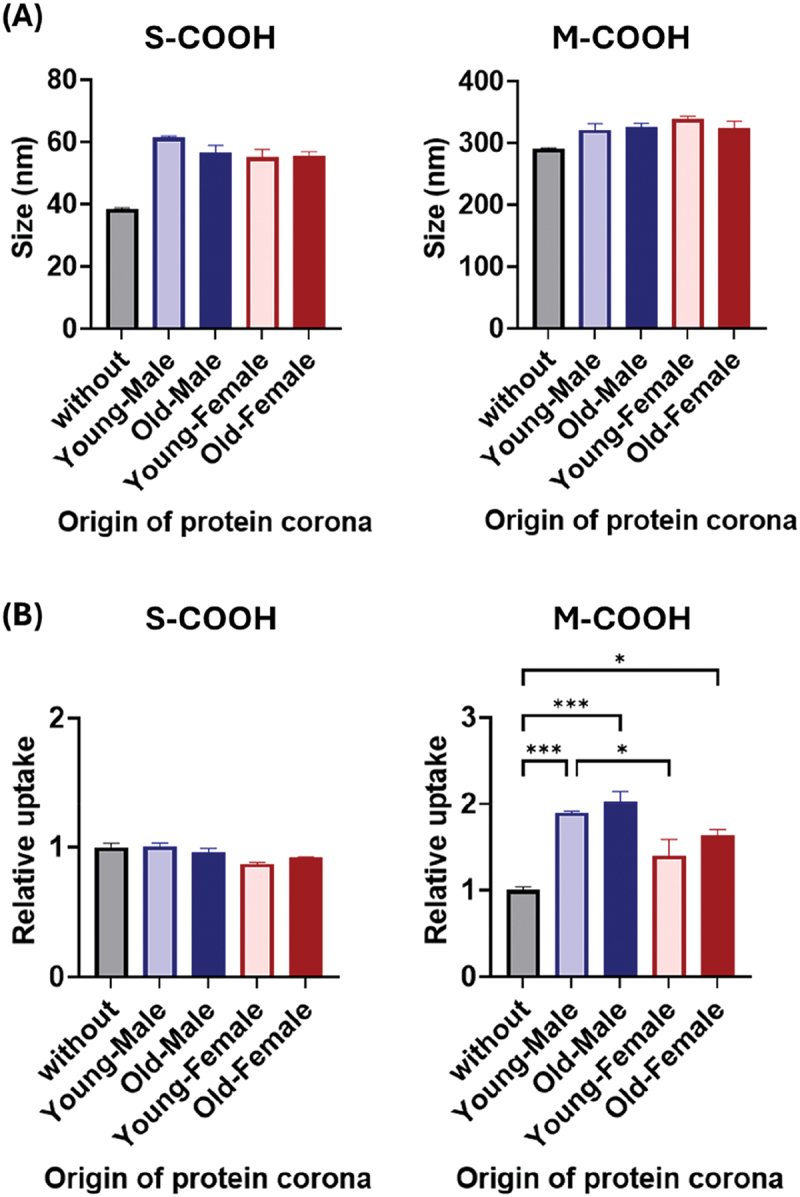
Modulation of nanoparticle uptake by protein corona. Prior to exposing RAW 264.7 mouse macrophages, nanoparticles were incubated with specified mouse serum to form protein coronas on their surfaces. (A) particles sizes (*n* = 3). (B) particle uptake (*n* = 3). Relative particle uptake is expressed as 1 for the uptake of nanoparticles without protein corona. Data are means ± SE. Statistical significance was determined by one-way ANOVA followed by Tukey’s test. *p < 0.05; ***p < 0.001.

Protein coronas (or biomolecule coronas) spontaneously form on the surfaces of particles when exposed to biological fluids, affecting their uptake [2]. Therefore, we sought to investigate the effects of mouse age and sex on the coronas formed on synthetic particle surfaces, as well as their impact on macrophage uptake. Serum samples from mice from each group were mixed with particle dispersions to form protein coronas on their surfaces. Formation of the protein corona layer increased the size of S-COOH and M-COOH particles by 17–23 nm (45%–60% increase) and 30–45 nm (10%–15% increase) compared with the corresponding original particles, respectively (Figure 6(A)). This observation aligns with previous findings that larger particles adsorb more total protein than smaller ones, but because of higher surface curvature, smaller particles tend to exhibit relatively greater percentage increases in sizes upon protein corona formation [44].

RAW264.7 macrophages were chosen to specifically isolate and evaluate the effects of protein-corona differences, while avoiding the confounding variations in intrinsic phagocytic capacity observed in primary BMMs due to age- and sex-dependent differences (Figure 2). This cell model thus provides a stable and reproducible baseline for assessing how serum-derived coronas influence nanoparticle uptake, independent of the biological variability inherent to primary macrophages. Covering M-COOH particles with the protein coronas enhanced their uptake efficacy up to twice that of the bare form. Protein coronas of male mice exerted a more pronounced effect than those of female mice, whereas no age-related differences were observed (Figure 6(B)). In contrast, macrophages showed similar uptake of S-COOH particles even after protein corona coverage. This is most likely due to the significantly lower amount of protein corona in S-COOH particles than in M-COOH particles, which limits the effect on receptor recognition.

## 4. Discussion

The present study demonstrated that particle-uptake ability is lower in older BMMs and female BMMs compared with their counterparts. Furthermore, in female mice, the protein corona has a weaker effect on increasing uptake compared with male mice, and the effect of age was limited in both sexes. In vivo, nanoparticles administered into the bloodstream accumulate mainly in the liver and spleen and are gradually eliminated from the circulatory system by phagocytes (e.g. macrophages, neutrophils) that are abundant in these organs. Studies on sex-based differences in nanoparticle biodistribution have shown that female rodents tend to have slower blood clearance rates for various types of nanoparticles, such as metal nanoparticles and liposomes [45,46]. However, reports on polymeric particles are extremely limited. With aging, the accumulation of nanoparticles in the liver, spleen, and lung decreases, leading to prolonged circulation time [4,47,48]. Specifically, in mice aged 50–60 weeks, expression of the MARCO scavenger receptor in hepatic macrophages is lower than that in young mice (6–8 weeks old), resulting in reduced accumulation in the liver and slower clearance from the blood [4]. Consistently, our results also revealed decreased *Marco* expression in older macrophages (22‒26 months old) in both sexes. Furthermore, blocking the MARCO receptor significantly decreased particle uptake, with the inhibitory effect being more pronounced in younger macrophages.

Previous studies on macrophage uptake used cell lines and primary macrophages (usually from young mice) to identify endocytosis or phagocytosis receptors for engineered particles and pathogens. For instance, these receptors include scavenger receptors (e.g. MARCO), mannose receptors (e.g. CD206), pattern recognition receptors (e.g. Toll-like receptors), C-type lectin receptors (e.g. Dectin), and complement receptors (CRs) [6,27,49–52]. In the present study, comparisons between the primary BMMs of different ages and sexes revealed differential expression in the MARCO receptor, which partially accounts for differences in macrophage-uptake ability. Other receptor families were not included in the DEGs. However, it is possible that protein expression of those receptors differs among macrophages of different ages and sexes [53,54]; therefore, potential associations with other receptors are not completely ruled out by this study.

Although differences in uptake ability are often discussed with respect to the interaction between particles (ligands) and their receptors, our results provide another perspective on the involvement of age and sex differences in the later stage of the uptake process. Appropriate expression or activity of actin and actin machinery components is necessary for successful particle uptake [55–57]. A recent report has shown that BMMs in older individuals ( >50 years old) have reduced phagocytic ability and less actin expression (in protein levels) compared with those in their younger counterparts (18–30 years old) [10]. We also identified age-dependent DEGs involved in cytoskeleton reorganization, albeit in limited numbers between old and young BMMs. Sex-dependent DEGs included more genes related to actin and cytoskeleton reorganization than age-dependent DEGs. At the same time, ECM and ECM-binding receptor genes were identified as sex-dependent DEGs, among which integrin α11βX and DDR have been reported to also function as mechano-sensing receptors [37–39]. Studies have shown that mechanical stimuli arising from interactions between ECM and its receptors significantly influence cell adhesion and the actin-mediated cytoskeleton reorganization, ultimately determining particle internalization [58,59]. Prime examples are integrins αMβ2 and αXβ2, which are important in mechanically activated phagocytosis [60,61]. In this process, talin binds activated integrins to actin filaments, and vinculin reinforces this binding, forming a mechanosensitive molecular clutch that transmits extracellular forces to the cytoskeleton [59–61]. Integrin α11βX has a similar mechanism, although its relationship with uptake capacity remains undetermined [37,38]. DDR is a nonintegrin receptor and receptor tyrosine kinase that, upon binding to collagen, activates downstream signaling cascades involving the dynamics of the actin cytoskeleton (e.g. RhoA and Rac1) [39]. Collectively, lower uptake ability in female BMMs is likely associated with an overall difference in components related to cytoskeleton reorganization, such as mechano-sensing receptors and actin machinery. Nevertheless, future studies are essential to determine the roles of the identified DEGs in macrophage particle uptake.

Compositions of serum proteins vary with mouse age and sex and may influence uptake by macrophages [13,62–69]. Among these, complement proteins (most commonly C3, C4, and C5) and immunoglobulins G (IgG) are well known as major components in the formation of protein coronas and accelerate engulfment by phagocytes (a process termed ‘opsonization’) [70]. Specifically, foreign particles covered with complement proteins or IgG are recognized by macrophages via CRs or Fc receptors, respectively. Furthermore, complement proteins can bind to the Fc region of IgG, re-mask particles precoated with IgG, and form complement-IgG complexes on the particles [70,71]. Importantly, these complexes act as a more potent promoter of phagocytosis than does complement or IgG alone [72,73]. Existing reports suggest that complement proteins are more abundant in males in both mice and humans, while their age-dependent changes in the blood are unclear in mice [13, 63–66]. In contrast, in mice, serum IgG concentrations generally increase with age, whereas higher values in male mice have been observed at specific ages ( >18-months-old) and in specific IgG subclasses (IgG2b and IgG3) [13,67,68]. The uptake of M-COOH particles exposed to mouse serum broadly correlates with serum complement concentrations and, to a lesser extent, with age. As mentioned earlier, complement binds to IgG-precoated particles to form complement-IgG complexes. Therefore, it is conceivable that the difference in serum complement concentrations between male and female mice is one of the factors explaining the difference in macrophage uptake of corona-covered M-COOH particles. Although it is beyond the scope of this study, further characterization of protein corona formed on the particles is warranted.

## 5. Conclusions

Our findings highlight the critical role of host-specific factors, namely age and sex, in modulating macrophage uptake of both engineered and biological particles. While physicochemical particle properties such as size and surface charge remain key determinants of cellular internalization, these parameters were significantly influenced by the host immune milieu. For the engineered particles tested, macrophages from younger mice exhibit higher uptake activity, with the reduced phagocytic activity in females being particularly evident in the young age group. A comparable pattern was observed for the bioparticles, including *E. coli*, *S. aureus*, and zymosan, where uptake declined with aging and was lower in females across all ages. RNA-seq analysis revealed age- and sex-dependent DEGs, including genes related to a scavenger receptor, mechano-sensing receptors, and actin cytoskeleton machinery. Among them, MARCO, a scavenger receptor, contributed to the age-dependent decline in uptake for engineered particles, as confirmed by antibody blocking. Moreover, sex-specific variations in compositions of serum protein corona further affect interactions at the particle – cell interface. Collectively, these results emphasize the necessity of incorporating host age and sex as critical design parameters in the development of materials for immunological and therapeutic applications. Such consideration could aid in the rational design of next-generation, personalized, and population-inclusive nanomedicine, and thus potentially improve efficacy and safety profiles. Nevertheless, the roles of DEGs related to mechano-sensing receptors and actin machinery in uptake differences warrant further study.

## Supplementary Material

Supplemental Material

## Data Availability

The data that support the findings of this study are available from the corresponding author upon reasonable request.
