# Supplementary material for: Distinct macrophage uptake of engineered and biological particles driven by host age and sex
Source: Sci Technol Adv Mater. 2026 Jan 12;27(1):2610875. doi: 10.1080/14686996.2025.2610875 (PMC12857665; doi:10.1080/14686996.2025.2610875)
Supplement: Supplemental Material [file TSTA_A_2610875_SM8727.docx]

Supporting Information

Distinct macrophage uptake of engineered and biological particles driven by host age and sex

Riki Toita^a, 1, *^, Yuki Shimizu^b, 1^, Jeong-Hun Kang^c^

^a^ Molecular Biosystems Research Institute, National Institute of Advanced Industrial Science and Technology (AIST), Ikeda, Osaka, Japan; ^b^ Molecular Biosystems Research Institute, National Institute of Advanced Industrial Science and Technology (AIST), Tsukuba, Ibaraki, Japan; ^c^ National Cerebral and Cardiovascular Center Research Institute, Suita, Osaka, Japan; ^1^ These authors contributed equally to this study.

*Corresponding author: Riki Toita, Ph.D.

Molecular Biosystems Research Institute, National Institute of Advanced Industrial Science and Technology (AIST), Osaka, Japan.

E-mail: toita-r@aist.go.jp


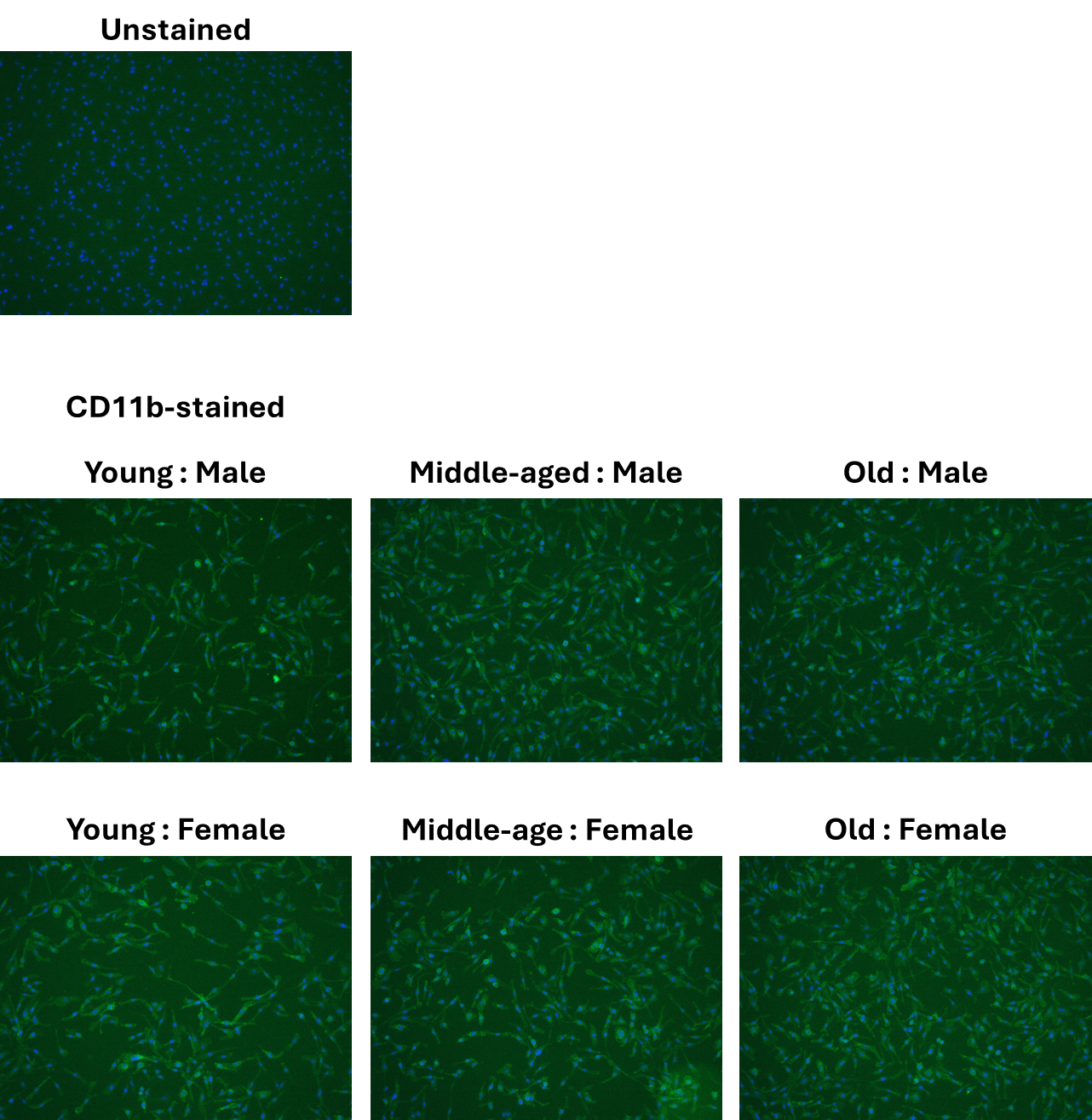


**Figure S1.** CD11b immunostaining of bone marrow-derived macrophages. After fixation with 4% paraformaldehyde in PBS, cells were stained with an Alexa Fluor 488–conjugated anti-mouse/human CD11b antibody (clone M1/70; 1:100; BioLegend, San Diego, CA, USA), and nuclei were counterstained with Hoechst 33342 (1:500; Dojindo, Kumamoto, Japan). Images were acquired using a ZOE Fluorescence Cell Imager (Bio-Rad Laboratories, Hercules, CA, USA). Brightness was adjusted uniformly across all images using PowerPoint.


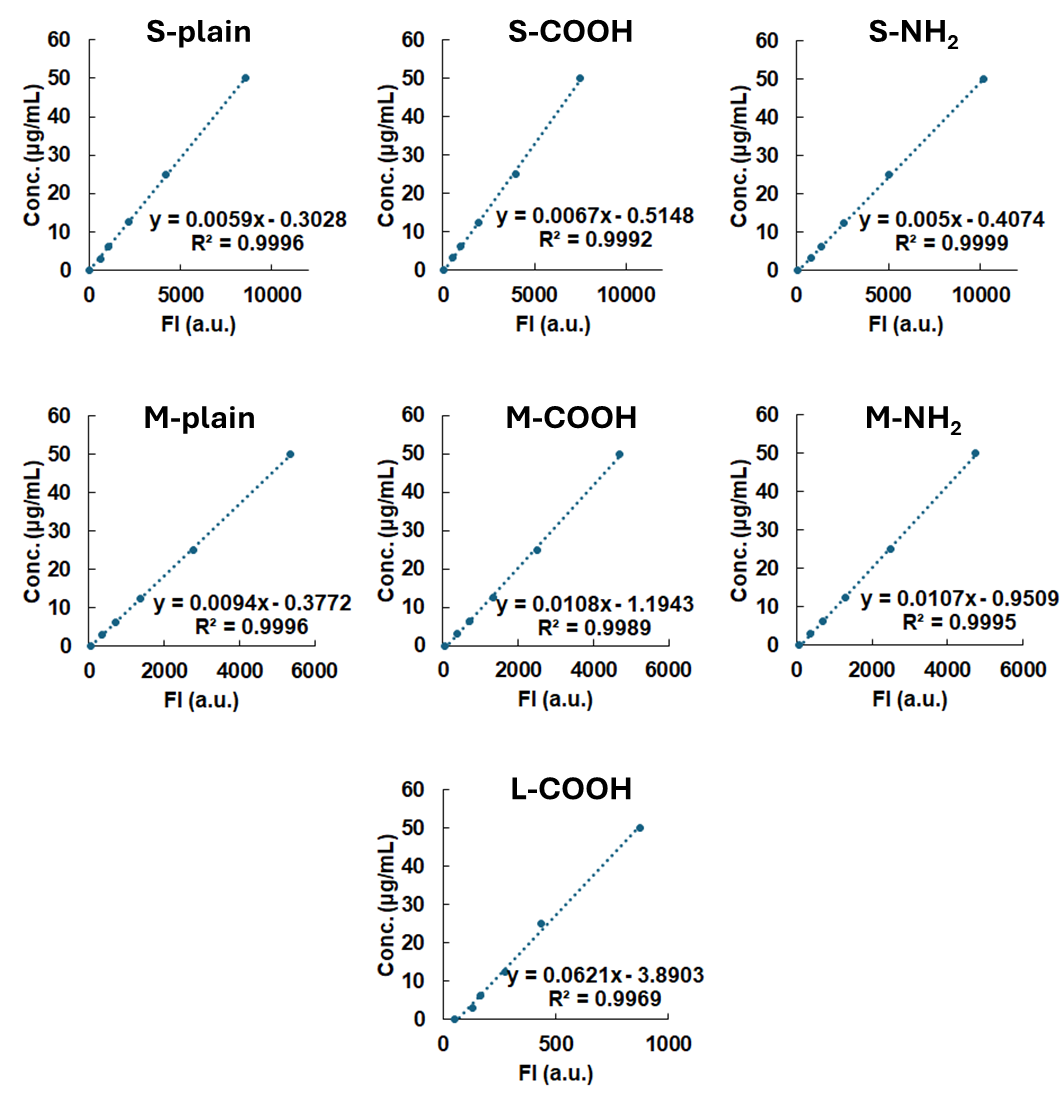


**Figure S2.** Standard slopes of fluorophore-modified polymeric particles.


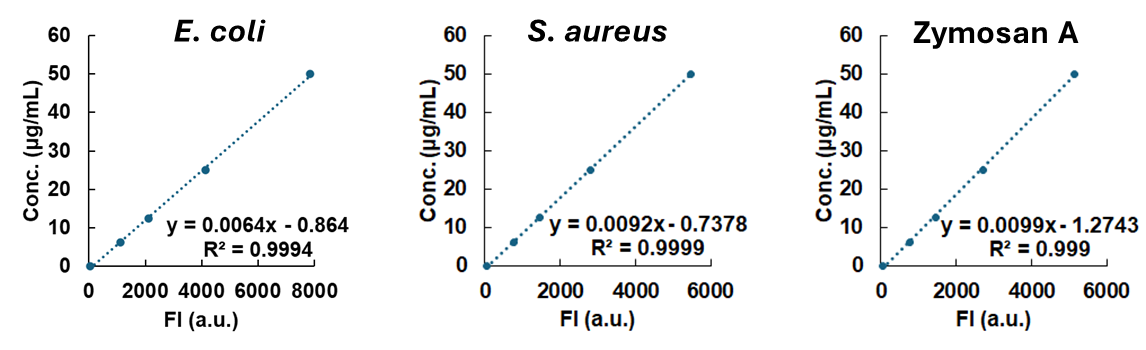


**Figure S3.** Standard slopes of fluorescein-modified bioparticles.

**
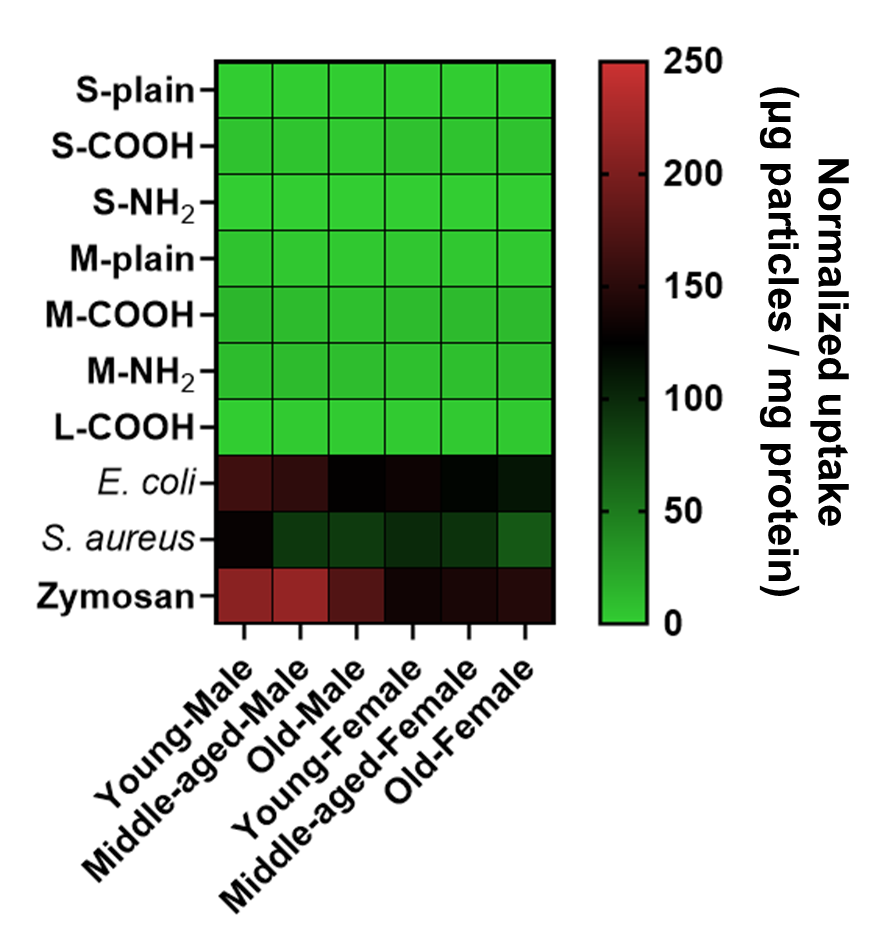
**

**Figure S4.** Amount of particle uptake 4 h after exposure of macrophages to particles.

**
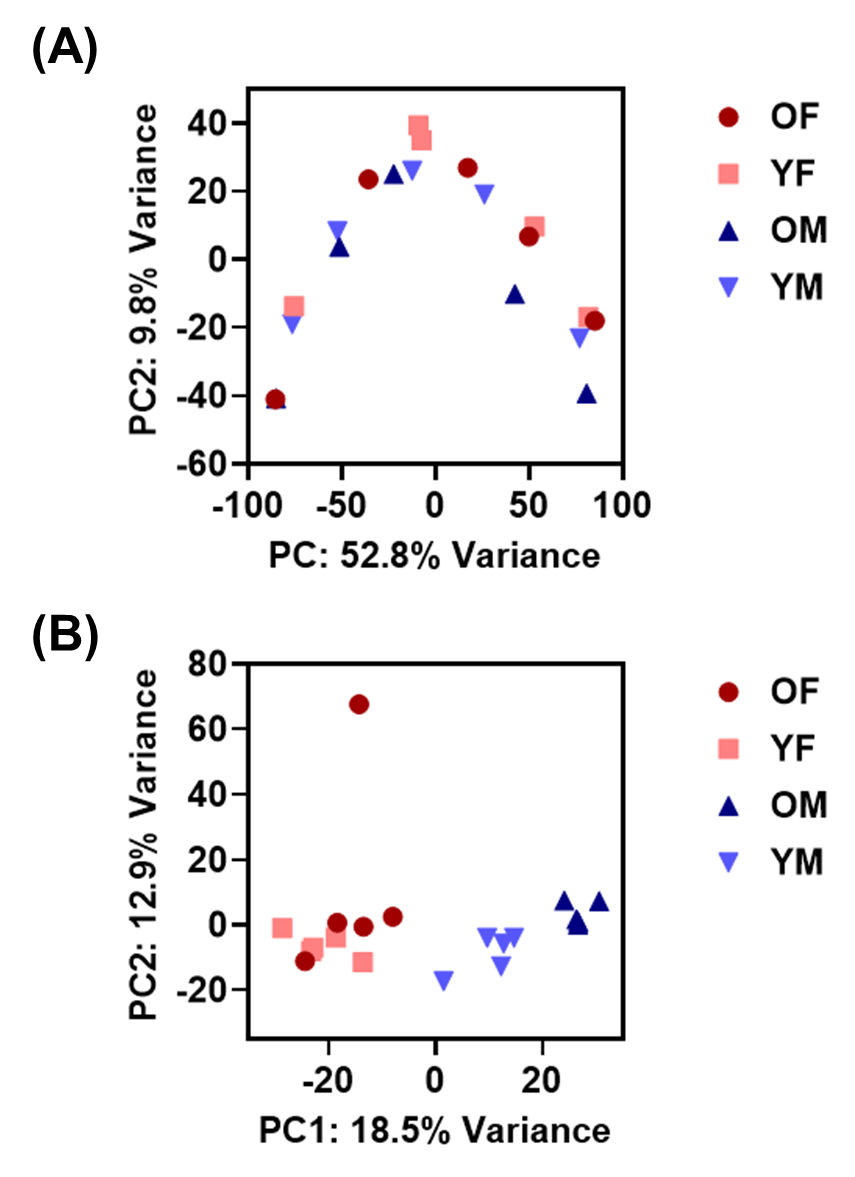
**

**Figure S5.** Principal component analysis (PCA). (A) PCA of raw count data before batch correction. (B) PCA after batch correction using ComBat-seq, showing mitigation of batch effects across samples and identification of a potential outlier sample.


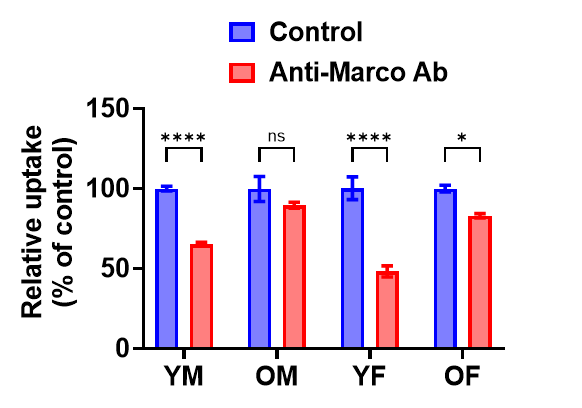


**Figure S6.** Blocking the MARCO receptor with an anti-MARCO antibody reduces the uptake of L-COOH particles by bone marrow-derived macrophages. Y, young; O, old; M, male; F, female. Data are means ± SE (*n* = 5). Statistical significance was determined by paired *t*-test. **p* < 0.05; *****p* < 0.0001; ns, not significant.

**Table S1.** List of common DEGs showing sex-dependent differences in macrophages.

| **High in female**  **(8 genes)** | **Low in female**  **(83 genes)** |  |  |
| --- | --- | --- | --- |
| *C1qtnf6* | *Eif2s3y* | *Col5a1* | *Cnn3* |
| *Pde1c* | *Ddx3y* | *Col16a1* | *Thbs2* |
| *Eif2s3x* | *Kdm5d* | *Sema7a* | *Col8a1* |
| *Padi4* | *Uty* | *Map1b* | *Amotl2* |
| *Odf3l1* | *Gm29650* | *Phldb2* | *Col12a1* |
| *Cpne8* | *ENSMUSG00000121547* | *Col3a1* | *Fn1* |
| *Gdf3* | *Grem2* | *Cyp1b1* | *Timp3* |
| *Xist* | *Cd248* | *Sparc* | *Fermt2* |
|  | *Vgll3* | *Col5a2* | *Ccn2* |
|  | *Ccn5* | *Cxcl12* | *Grb10* |
|  | *Eml1* | *Acta2* | *Dpysl3* |
|  | *Loxl1* | *Fstl1* | *Lamb1* |
|  | *Nid1* | *Col6a1* | *Tgfb1i1* |
|  | *Tjp1* | *Col5a3* | *Mxra8* |
|  | *Itga11* | *Loxl2* | *Parva* |
|  | *Col1a1* | *Fat1* | *Cemip* |
|  | *Actg2* | *Fkbp10* | *Fscn1* |
|  | *Hspg2* | *Fmod* | *Msrb3* |
|  | *Postn* | *Mrc2* | *Aebp1* |
|  | *Ddr2* | *Bgn* | *Bmp1* |
|  | *Col6a3* | *Serpinh1* | *Tead1* |
|  | *Igfbp7* | *Adam12* | *Ccdc80* |
|  | *Pxdn* | *Fbn1* | *Enah* |
|  | *Crlf1* | *Cald1* | *Timp1* |
|  | *Col1a2* | *Myl9* | *Tnc* |
|  | *Podnl1* | *Ccn4* | *Ltbp1* |
|  | *Fbln2* | *Cdh11* | *Inhba* |
|  | *Cdh2* | *Adamts2* |  |
